# Lead-Induced Motor Dysfunction Is Associated with Oxidative Stress, Proteome Modulation, and Neurodegeneration in Motor Cortex of Rats

**DOI:** 10.1155/2021/5595047

**Published:** 2021-10-07

**Authors:** Luana Ketlen Reis Leão, Leonardo Oliveira Bittencourt, Ana Carolina Alves Oliveira, Priscila Cunha Nascimento, Maria Karolina Martins Ferreira, Giza Hellen Nonato Miranda, Railson de Oliveira Ferreira, Luciana Eiró-Quirino, Bruna Puty, Aline Dionizio, Sabrina Carvalho Cartágenes, Marco Aurelio M. Freire, Marília Afonso Rabelo Buzalaf, Maria Elena Crespo-Lopez, Cristiane Socorro Ferraz Maia, Rafael Rodrigues Lima

**Affiliations:** ^1^Laboratory of Functional and Structural Biology, Institute of Biological Sciences, Federal University of Pará, Belém, Pará, Brazil; ^2^Department of Biological Sciences, Bauru Dental School, University of São Paulo, Bauru, São Paulo, Brazil; ^3^Laboratory of Pharmacology of Inflammation and Behavior, Pharmacy Faculty, Institute of Health Sciences, Federal University of Pará, Belém, Pará, Brazil; ^4^Graduate Program in Health and Society, University of the State of Rio Grande do Norte, Mossoró, Rio Grande do Norte, Brazil; ^5^Laboratory of Molecular Pharmacology, Institute of Biological Sciences, Federal University of Pará, Belém, Pará, Brazil

## Abstract

Lead (Pb) is a toxic metal with great neurotoxic potential. The aim of this study was to investigate the effects of a long-term Pb intoxication on the global proteomic profile, oxidative biochemistry and neuronal density in motor cortex of adult rats, and the possible outcomes related to motor functions. For this, Wistar rats received for 55 days a dose of 50 mg/Kg of Pb acetate by intragastric gavage. Then, the motor abilities were evaluated by open field and inclined plane tests. To investigate the possible oxidative biochemistry modulation, the levels of pro-oxidant parameters as lipid peroxidation and nitrites were evaluated. The global proteomic profile was evaluated by ultraefficiency liquid chromatography system coupled with mass spectrometry (UPLC/MS) followed by bioinformatic analysis. Moreover, it was evaluated the mature neuron density by anti-NeuN immunostaining. The statistical analysis was performed through Student's *t*-test, considering *p* < 0.05. We observed oxidative stress triggering by the increase in malonaldehyde and nitrite levels in motor cortex. In the proteomic analysis, the motor cortex presented alterations in proteins associated with neural functioning, morphological organization, and neurodegenerative features. In addition, it was observed a decrease in the number of mature neurons. These findings, associated with previous evidences observed in spinal cord, cerebellum, and hippocampus under the same Pb administration protocol, corroborate with the motor deficits in the rats towards Pb. Thus, we conclude that the long-term administration to Pb in young Wistar rats triggers impairments at several organizational levels, such as biochemical and morphological, which resulted in poor motor performance.

## 1. Introduction

Lead is one of the most abundant elements found on Earth, present as metallic form, lead salts, and organic lead [1–3]. The Institute for Health Metrics and Evaluation (IHME) estimated that, only in 2017, lead exposure accounted for 1.06 million deaths, with the highest burden impacting low- and middle-income countries [4]. Human exposure occurs through occupational process, drinking water, traffic pollution, lead extraction, coal burning, and also environment due to the increased lead levels on ice and soil in some regions [5–7]. In addition, lead is often used in pesticides and fertilizers, gasoline, battery pigments, cosmetics, and metal products such as ammunition, solders, and plumbing pipes [8–10]. This metal is known as an important environmental pollutant, non-biodegradable, and with toxic effects frequently reported, turning it into a serious public health problem [11].

Recently, we investigated the effects of long-term exposure to lead from adolescence to adulthood on different regions of the Central Nervous System (CNS) [12–14]. In the hippocampus, an important region involved in cognitive processes, we observed increased levels of lead, oxidative stress, and altered modulation of proteins related to cell protection, synaptic transmission, associated with intense neuronal loss, and damages to cognitive functions [14]. Our data also showed high levels of lead on spinal cord and cerebellum, and after long-term exposure, a reduction of neurons density in both regions was associated with motor changes [12, 13]. In fact, even these results showing toxic effects of lead in CNS, the information about the toxic effects on other areas of brain, such as the motor cortex, mainly in stages of development, are rare.

Considering the brain regions responsible for movement control, the motor cortex is responsible for motor planning and programming, in addition to command both from the spinal cord and brainstem that modulate reflexes and gross movements [15, 16]. Thus, the better understanding of the effects of lead exposure on the motor cortex can elucidate how lead can affect motor control. Therefore, the aim of this study was to investigate the effects of long-term administration to lead from adolescence to adulthood, on the motor cortex of rats, examining oxidative balance, proteomic profile, neuronal degeneration, and motor functions.

## 2. Materials and Methods

### 2.1. Animals and Experimental Design

This project was approved by the Ethics Committee from Federal University of Pará with protocol number 2237110716, following the NIH Guide for the Care and Use of Laboratory Animals. For this, we used 50 male Wistar rats (*Rattus norvegicus*), with 40 days old, weighing between 150 and 160 g. The animals were randomly divided into two groups, control and treated, with different numbers of animal in each group for the different analyses. In the treated group, it was administered a daily dose of 50 mg/kg of lead acetate (Sigma-Aldrich, St Louis, MO, USA) for 55 consecutive days. The control group received distilled water (the same proportional volume) by intragastric gavage. During the intoxication period, water and food were given *ad libitum*, and the animals were weekly weighed to dose adjustment.

**Figure 1:**
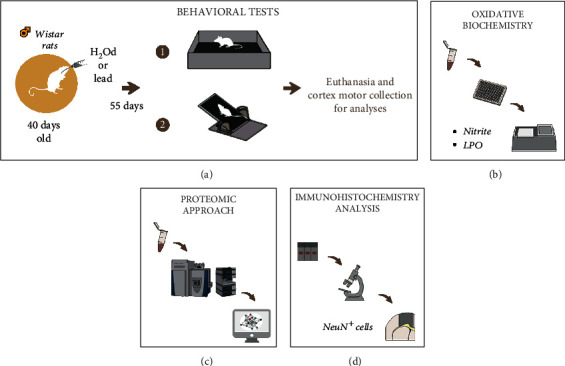
Sample description and experimental steps: (a) sample description (*n* = 25 per group) and lead administration protocol. After the administration period to lead acetate, the behavioral evaluation through (1) open field and (2) inclined plane tests (25 animals per group). Then, the euthanasia and brain collection for analyses in motor cortex: (b) oxidative biochemistry analyses through nitrite levels (nitrite) and lipid peroxidation (LPO), with 10 animals per group; (c) proteomic profile performed by mass spectrometry with 9 animals per group; (d) immunohistochemistry analysis by anti-NeuN (NeuN^+^ cells) in M1 area of motor cortex with 6 animals per group.

This lead administration protocol was based on Gu et al. [17] and reproduced in previous studies from our group [12–14, 18]. The experimental design is summarized in Figure 1.

### 2.2. Behavioral Tests

Twenty-four hours after the administration period, all the animals were conducted to the assay room with attenuated sounds and controlled illumination, in order to avoid stress during the habituation and behavioral procedures.

#### 2.2.1. Open Field

This behavioral assay investigated the spontaneous locomotor activity [15]. The animals were individually placed in the center of the apparatus, which consists of an acrylic box (100 × 100 × 30 cm) virtually divided into 25 quadrants, with free exploration for 5 minutes. All sessions were recorded and posteriorly analyzed by ANY-maze (Stoelting Co., Wood Dale, IL, USA) software. The parameters analyzed were total distance traveled, distance traveled on peripheral area, and distance traveled on the center. Following the open field test, the animals were submitted to the inclined plane assay.

#### 2.2.2. Inclined Plane

This test was performed according to a previous protocol [19]. The animals were placed on the 0° angled movable platform, which is composed of two rectangular platforms [20]. One platform is fixed, and the other one is angled movable (Insight, São Paulo, Brazil). The movable platform was covered with rubber to ensure animal adhesion and movement. The inclination angle was gradually increased from zero up to the highest inclination in which each animal was able to remain positioned for 5 seconds (every 5 seconds the angle was increased by 5 degrees) for 5 consecutive trials, with an intersession interval of 60 seconds, and the average angle was measured.

### 2.3. Oxidative Biochemistry Analyses

At the end of the behavioral tests, the animals from each group were randomly divided to the different analyses and then euthanized. Firstly, the animals (*n* = 10/per group) were anesthetized with a solution of ketamine hydrochloride (90 mg/kg, *i.p*) and xylazine hydrochloride (10 mg/kg, *i.p*) and then euthanized. The motor cortex was collected, frozen in liquid nitrogen, and stored at -80°C until further analyses. Then, the samples were thawed, resuspended in Tris-HCl buffer (20 mM, pH 7.4), and disaggregated sonically. The samples were separated into two aliquots: for nitrite and lipid peroxidation determinations. The detailed protocol for nitrite measurements was previously established by Green et al. [21] and for lipid peroxidation, we used the method proposed by Esterbauer and Cheeseman [22]. Both results were normalized by protein concentration by Bradford's method [23].

### 2.4. Proteomic Approach

All proteomic analyses were performed according to protocols previously described elsewhere [24, 25]. Nine animals per group were euthanized and used in the proteomic approach. The samples of motor cortex from two animals were pooled, and all the procedures were carried out in triplicate. Briefly, the proteomic consists of protein extraction by lysis buffer. Then, the samples were reduced, alkylated, and finally digested by trypsin and desalted by C18 spin column (Pierce, Thermo Fisher, USA). Afterward, the samples were resuspended in the solution containing 12 *μ*L of alcohol dehydrogenase standard (1 pmol/*μ*L) + 108 *μ*L of 3% acetonitrile and 0.1% formic acid.

The reading and identification of the peptides were performed on a nanoAcquity UPLC-Xevo QTof MS system (Waters Corporation, Wilmslow, UK), which were interpreted by Protein Lynx Global Server (PLGS) software applying the Monte-Carlo algorithm. After comparing the experimental groups, it was considered *p* < 0.05 for downregulated proteins and 1 − *p* > 0.95 for upregulated proteins. It was used the *Rattus norvegicus* proteome downloaded from Uniprot. After, the proteins identified were analyzed by a bioinformatic approach using Cytoscape 3.6.1 (Java®) with ClueGO plugin [26].

### 2.5. Histological Procedures

To evaluate morphological changes in the motor cortex, the animals (*n* = 6/per group) were anesthetized with a solution of ketamine hydrochloride (90 mg/kg *i.p.*) and xylazine hydrochloride (10 mg/kg, *i.p.*) and perfused through the left ventricle of the heart with solution heparinized 0.9% saline, followed by 4% paraformaldehyde. The samples were postfixed in Bouin solution for 6 hours and then processed and embedded in paraplast (McCormick Scientific, St Louis, MO, USA) [27].

After inclusion, the samples were sectioned by a microtome to obtain sections with 5 *μ*m of thickness. All histological analyses were performed in M1 area of motor cortex, specifically between layers 3 and 4 (pyramidal neurons areas), through the coordinates: 2.5 mm lateral, 1.2 mm posterior, and 4.5 mm below from the pial surface (at Bregma 0.20 mm and interaural 9.20 mm) [28].

#### 2.5.1. Immunohistochemical Analysis

The slides with sections were dewaxed and immersed in PBS for 3 min before antigen retrieval in citrate buffer at 70°C for 25 min. Afterward, the sections had endogenous peroxidase inhibited by immersing in methanol-hydrogen peroxide solution (3%). We used anti-NeuN antibody (1 : 100, Chemicon) for immunolabeling of mature neurons [15, 29, 30]. We proceeded the revelation was 3,3′-diaminobenzidine solution in PBS and coverslipped with Entellan® (Merck, Darmstadt, Germany) [27, 31, 32]. The positive cells for anti-NeuN immunostaining were analyzed by light microscopy (Nikon Eclipse E200, Tokyo, Japan) with a 0.0625 mm^2^ grid attached to the ocular and using objective lens of 40×, evaluating the density of anti-NeuN+ cells [15]. The photomicrographs were obtained using the microscope Nikon Eclipse E500 with Moticam 2500® attached to it.

### 2.6. Statistical Analyses

Results were tabulated and analyzed by GraphPad Prism 7.0 software (GraphPad Software Inc., La Jolla, CA, USA), and the Shapiro Wilk normality test was performed. The hypotheses were tested by Student's *t*-test. In order to evaluate the body mass gain over the time, we applied the repeated measures two-way ANOVA test. The level of significance was set at *p* < 0.05.

## 3. Results

### 3.1. Long-Term Lead Administration Did Not Affect the Body Weight Gain of Rats

**Figure 2:**
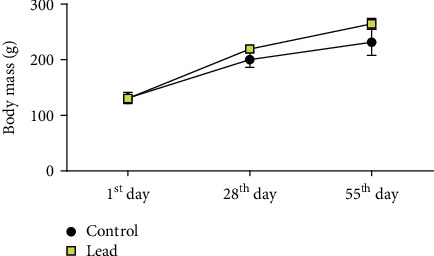
Body mass curve of experimental animals during the experimental period. Control group (black-filled circle) and treated group (yellow-filled square) from 1^st^ day to 55^th^ day of lead administration (50 mg/kg/day). The results are expressed as mean ± standard error of mean of body mass (g). Repeated measures two-way ANOVA test, *p* > 0.05. 25 animals per group.

At the end of the experiment, all animals increased the body mass as expected, and no difference between groups was observed (*p* > 0.05; Figure 2).

### 3.2. Long-Term Lead Administration Triggers Oxidative Stress on Motor Cortex of Adult Rats

**Figure 3:**
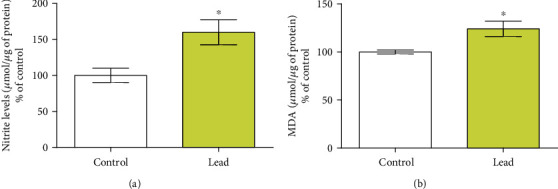
Effects of the long-term administration of lead acetate (50 mg/kg) after 55 days (*n* = 10 animals per group). (a) Nitrite levels and (b) malondialdehyde. Results are expressed (mean ± SEM) of percentages of nitrite per microgram of protein in relation to the control group and (B) percentages of milligram malondialdehyde per microgram of protein in relation to the control group. ^∗^Student *t*-test, *p* < 0.05.

The biochemical analyses showed that long-term administration of lead increased nitrite levels in motor cortex (*p* < 0.05, Figure 3(a)), as well as MDA levels (*p* < 0.05, Figure 3(b)).

#### 3.2.1. Long-Term Administration to Lead Modulates the Motor Cortex Proteomic Profile of Rats

After the long-term administration of lead, the motor cortex proteome was affected, which resulted in the modulation of several biological processes (BP). It was observed a total of 34 proteins that were downregulated and 239, upregulated in Pb group, 112 proteins exclusively expressed in the control group, and 87 unique in the Pb group (Supplementary Tables 2 and 3).

**Figure 4:**
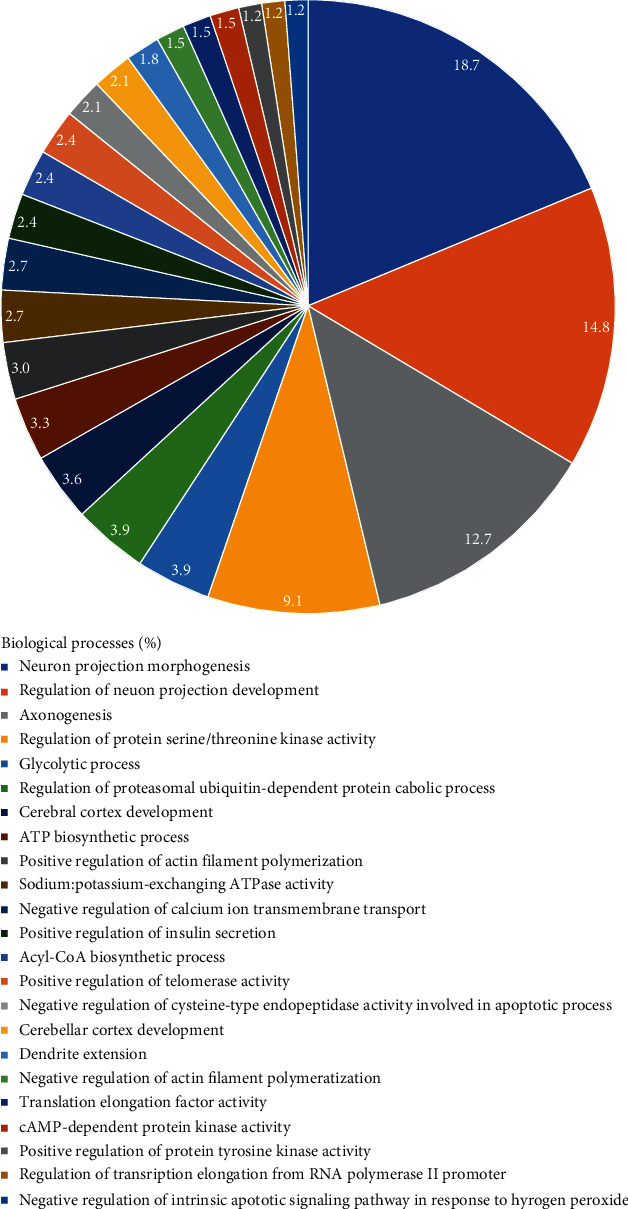
Functional distribution of proteins identified with differential expression in the motor cortex of rats administration of lead vs. control group. 9 animals per group. Categories of proteins based on Gene Ontology annotation of the biological process. Terms significant (kappa score = 0.4) and distribution according to the percentage of the number of genes. Protein access number was provided by UNIPROT. The gene ontology was evaluated by ClueGO® plugin of Cytoscape® 3.8.2.

Moreover, the bioinformatic analyses based on Gene Ontology identified 23 BP, in which the top five were neuron projection morphogenesis (18.7%), regulation of neuron projection development (14.8%), axonogenesis (12.7%), regulation of serine/threonine kinase activity (9.1%), glycolytic process (3.9%), and others, including others as cerebral cortex development (3.6%) and negative regulation of calcium ion transmembrane transport (2.7%) (Figure 4).

### 3.3. Long-Term Administration to Lead Decreases Mature Neurons Cells in Motor Cortex of Rats

**Figure 5:**
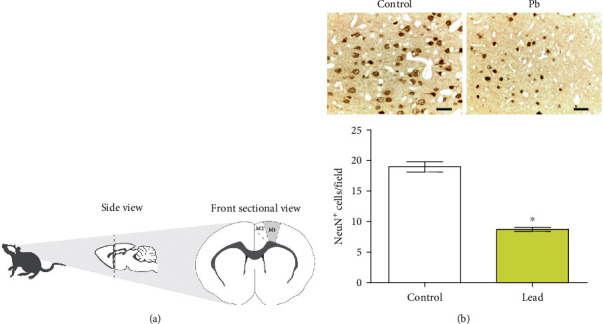
Effects of the long-term administration of lead acetate (50 mg/Kg) on M1 area (a) morphology in motor cortex of young Wistar rats (*n* = 6/animal per group). The results are expressed as mean ± standard error of mean of NeuN^+^ cells density (b). ^∗^Student *t*-test, *p* < 0.05. Scale bars: 30 *μ*m.

It was evidenced a decreased number of anti-NeuN^+^ cells in primary motor area (M1) from motor cortex of rats that received lead when compared to control group (*p* < 0.05; Figure 5).

### 3.4. The Accumulative Motor Cortex Damages Caused by Long-Term Administration to Lead Reflected on Poor Motor Performance in Adult Rats

**Figure 6:**
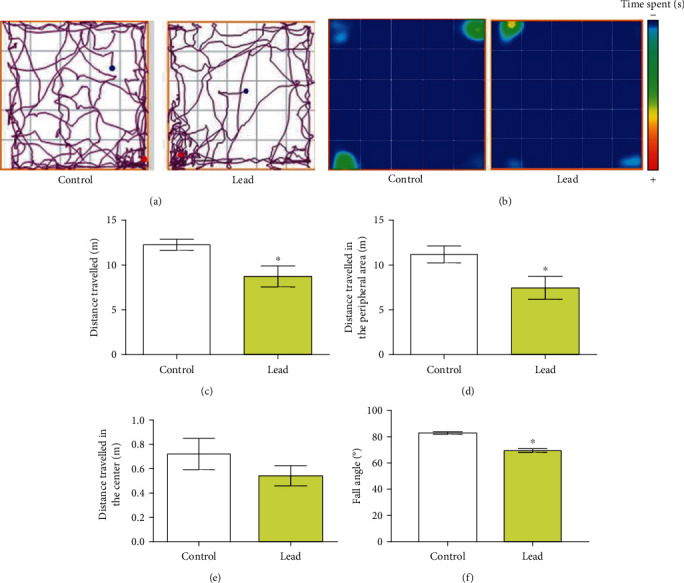
Effects of the long-term administration of lead acetate (50 mg/kg) on the exploratory activity and motor function of young Wistar rats (25 animals per group). The results are expressed as mean ± standard error of mean of open field and inclined plane tests. The animals' performance on open field test is represented by the (a) tracking plot and (b) heat-map, (c) total distance traveled, (d) distance traveled in the peripheral area, (e) distance traveled in the central area, and (d) fall angle in the inclined plane test. ^∗^Student *t*-test, *p* < 0.05.

Our results showed that lead long-term lead administration affected horizontal spontaneous locomotion (Figures 6(a) and 6(b)), observed by the decrease of the total distance traveled (Table S3, *p* = 0.0245, Figure 6(c)) and the distance traveled on the peripheral area (Table S3, *p* < 0.05, Figure 6(d)), while no changes on central distance traveled was observed (Supplementary Table 3, *p* > 0.05, Figure 6(e)). On the inclined plane assay, the lead-intoxicated animals also displayed poorer performance, reducing the fall angle parameter (Supplementary Table 3, *p* < 0.05, Figure 6(f)).

## 4. Discussion

This study reunites a combination of approaches that provide the characterization of biochemical, proteomic, and histological changes on motor cortex associated with locomotor deficits induced by lead long-term administration on adolescent rats. Our results showed that the lead challenge elicited oxidative stress, neurodegeneration and modulation of important proteins associated with synaptic communication, cell signaling, and survival, among others, potentially related to the motor skills impairments observed. These evidences reinforce out latest data regarding the metal effects over spinal cord and cerebellum, two others regions associated with higher motor command, unraveling new evidences about lead neurotoxicity.

The motor cortex consists of the brain region responsible for planning and spontaneous movement execution [33]. Although neural control of the movement starts on the prefrontal cortex and is modulated by the basal and cerebellar ganglia, the motor cortex consists of the main effector area for descending pathways and plays an essential function in fine motor control and fractionation of movement sensorimotor integration, besides acting in higher-order cognitive–motor movements [34–36]. Thus, considering that the motor cortex plays a fundamental role on motor function, this brain area has been extensively studied to evaluate its susceptibility to metal exposure [15, 37, 38].

Most part of studies about the effects of lead exposure on animal models are performed via drinking water [39]. However, although lead concentration on drinking water is not variable, it is not possible to assure the exact metal quantity that the host was exposed over time. In this way, orogastric gavage is a more reliable way since lead is administered directly on stomach. In addition, although we have not performed the determination of lead levels in motor cortex, the levels observed in the exposed groups in the previous works of our group with spinal cord [12] and cerebellum [13], associated with the changes observed herein, we suggest that there was a significant increase of the metal level in motor cortex.

In this perspective, considering the systemic distribution of lead after intragastric administration, the motor cortex is susceptible to changes on oxidative biochemistry, by increasing MDA and nitrite levels. The increase on lipid peroxidation, visualized by higher MDA levels, is associated with the oxidation of polyunsaturated lipids present in cell and organelles membrane by reactive oxygen species (ROS) [40] that may significantly affect the cell integrity and homeostasis, driving to cell death [41]. On the other hand, nitrites are indirect markers for oxide nitric (NO) due its short life that possesses several biological functions, but also acts as an oxidative stress mediator [42]. In addition, the proteomic revealed important components of redox balance impaired by lead administration, as the upregulation of *Glutathione S Transferase* (P04906), *Superoxide Dismutase* (P07632), *Peroxiredoxin 2* (P35704) and *5* (Q9R063), and unique expression of *Catalase* (P04762) in the control group. In this way, these data suggest that the breakdown of oxidative biochemistry homeostasis may contribute to lead toxicological mechanism of damage, also related to the proteomic alterations.

Looking to the proteomic approach, several proteins are indicative of a redox status imbalance, as the heat shock proteins (HSP), that have been present in several previous works from our group which investigated the effects of metals on CNS proteome [13, 14, 25, 43–46]. The lead administration up-regulated the expression of HSP subunits as 71 (P63018), 75 (Q5XHZ0), and 90 alpha (P82995) and beta (P34058). The HSP 70 (O35162) was found exclusively expressed in the Pb group. This family of chaperones exerts important roles in cells, as a mechanism of stress response, to avoid malfunction of protein folding, assembly, and transport, which could drive to protein function loss and aggregation [47, 48].

Associated to the discussion raised above, the energy metabolism process is intrinsically linked to both, oxidative stress triggering and further cell loss of function and death [49]. We observed the up-regulation of several ATP synthase subunits (P15999, P10719, P31399, Q06647, and P21571), *Citrate Synthase* (Q8VHF5), *Cytochrome C Oxidase subunits* (P00406, P11240, and P10818), *Pyruvate carboxylase* (P52873), and *Pyruvate dehydrogenase* (P26284) that are involved in glucose metabolism and affect the cell energy balance, suggesting an impairment on cell energy metabolism.

The failure on energy metabolism may impact directly the activity of neurons and glial cells due to its high demand [50, 51]. In this perspective, our proteomic approach suggests that lead triggers metabolic dysfunction that affects the up-regulation of proteins as *Excitatory amino acid transporter 1* (P24942) and *2* (P31596), besides the synaptic communication as shown by the up-regulation of *Syntaxin 1B* (P61265) and *Syntaxin-binding protein 1* (P61765), *Synasin 1* (P09951), and *2* (Q63537). In addition to that, the preservation of myelin sheath structure is important to the action potential, and it was observed an up-regulation of *CD9 antigen* (P40241), *Myelin Basic Protein* (P02688), and *Myelin-Associated Glycoprotein* (P07722); it was also observed the down-regulation of *Myelin Proteolipid Protein* (P60203). Considering these components, we suggest that neuroglial function might be compromised due to several issues in the background, as energy metabolism, oxidative stress, hence, synaptic communication and signal transduction.

Regarding the synaptic activity, the proteomic approach revealed the modulation of *Synatogyrin-1* (Q62876, unique in control group), that is, involved in short- and long-term synaptic plasticity [52, 53]. Moreover, it was observed the modulation of *Synapsin-1* and *2* (P09951 and Q63537, respectively) that are associated with neurotransmitter release [54], and some evidences correlate this protein with neurological disorders [55]. In this, we can hypothesize that lead affects the neurotransmission system and triggers motor impairments related to motor cortex observed in our behavioral tests.

Interestingly, it is worthy to point out that in previous proteomic data of hippocampus [14] after the same lead exposure model, the *Apolipoprotein E* (P02650, unique in control group) showed the same status of regulation in hippocampus, while *Protein S100B* (P04631) was up-regulated in motor cortex and down-regulated in hippocampus, on the other hand, these proteins were not changed in cerebellum [13]. However, the *CB1 cannabinoid receptor-interacting protein* 1 (Q5M7A7) found up-regulated in this study was exclusively expressed in the control group in cerebellar global proteomic profile. These are very distinct proteins involved mainly in lipid transport (P02650) [56], glial and neuronal trophic factor (P04631) [57], and neurotransmission processes (Q5M7A7) by interacting with CB1 cannabinoid receptor [58]. Although these proteins play different roles, they have been associated with neurological conditions of injuries and diseases [59–61], and synaptic plasticity [58] suggesting as important target proteins, and possible biomarkers, for new investigations regarding their status of regulation, anatomical region, and roles.

The literature has extensively described the mechanism of lead neurotoxicity related to the- interaction with Ca^2+^, and due to that, several processes as energy metabolism, cell signaling, neurotransmitter release, and apoptosis may be affected [62]. When looking for possible tissue changes that would show the repercussion of the biochemical (oxidative stress triggering) and proteomic changes found, our immunohistochemical analysis showed a reduction in the density of mature neurons in M1 area. Mature neurons in the motor cortex have been shown to be sensitive to other models of toxicological exposure to metals, such as exposure to inorganic mercury [37] and methylmercury [15, 16], and the reduction in density is associated with decreased spontaneous exploratory capacity, with impaired balance and motor coordination. The neurons present in primary motor area from motor cortex send outputs through corticofugal fibers of the pyramidal tract, which plays an important role in motor function. Moreover, this tract not only has a pivotal role in coordination of movement and posture but also is related to movements of eyes, limb musculature, and trunk, e.g., [63].

Our behavioral tests support the evidence that the neurodegeneration and modulation of synaptic communication proteins observed in the motor cortex were reflected in ethological behavior alteration, with a reduced spontaneous exploration profile. This first evidence is supported by the results of the open field test, in which the decrease in the total distance, with changes in the peripheral distance. It is noteworthy that the central distance traveled was not modified by lead administration, which reflects that the motor impairment observed was not accompanied of anxiogenic-like behavior. In addition, our results showed that lead administration decreased fall angle on the inclined plane task. Such result leads to other evidence of cortical damage and motor repercussion, with negative reflexes on the index of hind limb strength.

## 5. Conclusion

Considering all the changes observed in our study, we conclude that the long-term lead administration at a dose of 50 mg/kg/day causes poor motor performance associated with molecular and morphological impairments in motor cortex of adult Wistar rats. The lead administration triggered oxidative stress, modulated the global proteomic profile, including key proteins related to neurotransmission, cell metabolism, and signaling, leading to a motor function impairment.

## Data Availability

The quantitative and qualitative data used to support the findings of this study are included within the article and supplementary materials.

## Supplementary Materials

Supplementary Materials Lead-induced motor dysfunction is associated with oxidative stress, proteome modulation, and neurodegeneration in motor cortex of rats.
