# Supplementary material for: Lead-Induced Motor Dysfunction Is Associated with Oxidative Stress, Proteome Modulation, and Neurodegeneration in Motor Cortex of Rats
Source: Oxid Med Cell Longev. 2021 Oct 7;2021:5595047. doi: 10.1155/2021/5595047 (PMC8516562; doi:10.1155/2021/5595047)
Supplement: Supplementary Materials — Lead-induced motor dysfunction is associated with oxidative stress, proteome modulation, and neurodegeneration in motor cortex of rats. [file 5595047.f1.docx]

*Supplementary material*

**Lead-induced motor dysfunction is associated with oxidative stress, proteome modulation and neurodegeneration in motor cortex of rats**

This Supporting Information contains 3 tables.

| Behavioral parameters | | | |
| --- | --- | --- | --- |
| *Measure* | Control group | Lead group | p-value* |
| Open Field | | | |
| Total distance travelled (m) | 12.26 (± 0.62) | 8.78 (±1.1) | <0.05 |
| Distance Traveled in the peripheral area (m) | 11.15 (±0.93) | 7.49 (± 1.22) | <0.05 |
| Distance Traveled in the center (m) | 0.71 (±0.12) | 0.54 (±0.08) | >0.05 |
| Inclined plan | | | |
| Fall Angle (º) | 82.78 (±1.21) | 69.44 (±1.54) | <0.05 |
| Biochemistry oxidative parameters | | | |
| *Measure* | Control group | Lead group | p-value |
| Nitrite (µmol/µg of protein) - % of control | 100 (±9.99) | 160 (±17.44) | <0.05 |
| MDA (nmol/µg of protein) - % of control | 100 (±2.05) | 124.2 (±8.01) | <0.05 |
| Immunohistochemical analyses | | | |
| NeuN^+^ cells (n) | 19 (±0.83) | 9 (± 0.41) | <0.05 |
|  |  |  |  |

**Table S1.** Quantification data for all analysis carried out presented in mean and standard error (n=30 per group)

*Student’s t-test (p≤0.05)

| Table S2. Global proteomic profile of rats motor cortex exposed or not to lead acetate. List of proteins with differential regulation in comparison to control group. | | | | | | |  |
| --- | --- | --- | --- | --- | --- | --- | --- |
| Accession Id^a^ | **Protein Description** | **Score** | **Fold Change** | | |  |  |
| Q6P6V0 | Glucose-6-phosphate isomerase | 53818 | 3.63 | | |  |  |
| Q6AY30 | Saccharopine dehydrogenase-like oxidoreductase | 29936 | 3.49 | | |  |  |
| P16036 | Phosphate carrier protein, mitochondrial | 111681 | 3.35 | | |  |  |
| O35095 | Neurochondrin | 24866 | 3.12 | | |  |  |
| Q09073 | ADP/ATP translocase 2 | 169510 | 2.38 | | |  |  |
| O08984 | Delta(14)-sterol reductase LBR | 17809 | 2.27 | | |  |  |
| P11506 | Plasma membrane calcium-transporting ATPase 2 | 37015 | | 2.27 |  |  |  |
| P35738 | 2-oxoisovalerate dehydrogenase subunit beta, mitochondrial | 22446 | | 2.05 |  |  |  |
| P20651 | Serine/threonine-protein phosphatase 2B catalytic subunit beta isoform | 39939 | | 2.05 |  |  |  |
| P13638 | Sodium/potassium-transporting ATPase subunit beta-2 | 73900 | | 2.05 |  |  |  |
| Q9JHU0 | Dihydropyrimidinase-related protein 5 | 21112 | | 1.97 |  |  |  |
| P11505 | Plasma membrane calcium-transporting ATPase 1 | 37726 | | 1.97 |  |  |  |
| Q64568 | Plasma membrane calcium-transporting ATPase 3 | 17581 | | 1.85 |  |  |  |
| Q64541 | Sodium/potassium-transporting ATPase subunit alpha-4 | 103132 | | 1.84 |  |  |  |
| Q5M7U6 | Actin-related protein 2 | 29114 | | 1.82 |  |  |  |
| P97546 | Neuroplastin | 48874 | | 1.82 |  |  |  |
| P00406 | Cytochrome c oxidase subunit 2 | 310648 | | 1.80 |  |  |  |
| P54708 | Potassium-transporting ATPase alpha chain 2 | 66392 | | 1.80 |  |  |  |
| P06686 | Sodium/potassium-transporting ATPase subunit alpha-2 | 360616 | | 1.80 |  |  |  |
| O70351 | 3-hydroxyacyl-CoA dehydrogenase type-2 | 25359 | | 1.78 |  |  |  |
| P16290 | Phosphoglycerate mutase 2 | 33920 | | 1.78 |  |  |  |
| P52873 | Pyruvate carboxylase, mitochondrial | 44600 | | 1.78 |  |  |  |
| P09626 | Potassium-transporting ATPase alpha chain 1 | 50325 | | 1.76 |  |  |  |
| P11517 | Hemoglobin subunit beta-2 | 205874 | | 1.75 |  |  |  |
| P63329 | Serine/threonine-protein phosphatase 2B catalytic subunit alpha isoform | 102659 | | 1.73 |  |  |  |
| Q5BK63 | NADH dehydrogenase [ubiquinone] 1 alpha subcomplex subunit 9, mitochondrial | 54630 | | 1.71 |  |  |  |
| Q9R1Z0 | Voltage-dependent anion-selective channel protein 3 | 140115 | | 1.69 |  |  |  |
| P51635 | Aldo-keto reductase family 1 member A1 | 42220 | | 1.68 |  |  |  |
| P24942 | Excitatory amino acid transporter 1 | 17098 | | 1.64 |  |  |  |
| P61765 | Syntaxin-binding protein 1 | 841234 | | 1.63 |  |  |  |
| P04642 | L-lactate dehydrogenase A chain | 233439 | | 1.61 |  |  |  |
| O08557 | N(G),N(G)-dimethylarginine dimethylaminohydrolase 1 | 37257 | | 1.58 |  |  |  |
| P02688 | Myelin basic protein | 2971514 | | 1.55 |  |  |  |
| P01946 | Hemoglobin subunit alpha-1/2 | 3859366 | | 1.53 |  |  |  |
| P62762 | Visinin-like protein 1 | 30427 | | 1.53 |  |  |  |
| P84092 | AP-2 complex subunit mu | 121770 | | 1.52 |  |  |  |
| Q6URK4 | Heterogeneous nuclear ribonucleoprotein A3 | 24962 | | 1.50 |  |  |  |
| Q5U300 | Ubiquitin-like modifier-activating enzyme 1 | 33697 | | 1.50 |  |  |  |
| P08753 | Guanine nucleotide-binding protein G(i) subunit alpha | 255710 | | 1.49 |  |  |  |
| P63095 | Guanine nucleotide-binding protein G(s) subunit alpha isoforms short | 245351 | | 1.49 |  |  |  |
| Q6Q7Y5 | Guanine nucleotide-binding protein subunit alpha-13 | 258819 | | 1.49 |  |  |  |
| P63086 | Mitogen-activated protein kinase 1 | 19855 | | 1.49 |  |  |  |
| P97685 | Neurofascin | 20669 | | 1.49 |  |  |  |
| Q9QWN8 | Spectrin beta chain, non-erythrocytic 2 | 21473 | | 1.49 |  |  |  |
| P04897 | Guanine nucleotide-binding protein G(i) subunit alpha-2 | 360356 | | 1.47 |  |  |  |
| P07722 | Myelin-associated glycoprotein | 37993 | | 1.47 |  |  |  |
| P18484 | AP-2 complex subunit alpha-2 | 34364 | | 1.46 |  |  |  |
| Q07266 | Drebrin | 30362 | | 1.46 |  |  |  |
| P10824 | Guanine nucleotide-binding protein G(i) subunit alpha-1 | 290859 | | 1.46 |  |  |  |
| P38406 | Guanine nucleotide-binding protein G(olf) subunit alpha | 244619 | | 1.46 |  |  |  |
| P29348 | Guanine nucleotide-binding protein G(t) subunit alpha-3 | 255710 | | 1.46 |  |  |  |
| Q63210 | Guanine nucleotide-binding protein subunit alpha-12 | 254513 | | 1.44 |  |  |  |
| Q7TPB1 | T-complex protein 1 subunit delta | 29288 | | 1.44 |  |  |  |
| P07340 | Sodium/potassium-transporting ATPase subunit beta-1 | 841435 | | 1.41 |  |  |  |
| Q9Z1P2 | Alpha-actinin-1 | 44601 | | 1.40 |  |  |  |
| P55161 | Nck-associated protein 1 | 18387 | | 1.40 |  |  |  |
| Q9QUH6 | Ras/Rap GTPase-activating protein SynGAP | 19549 | | 1.40 |  |  |  |
| P40241 | CD9 antigen | 55069 | | 1.39 |  |  |  |
| P31016 | Disks large homolog 4 | 22910 | | 1.39 |  |  |  |
| Q63803 | Guanine nucleotide-binding protein G(s) subunit alpha isoforms XLas | 248851 | | 1.39 |  |  |  |
| P47942 | Dihydropyrimidinase-related protein 2 | 1997204 | | 1.37 |  |  |  |
| P39052 | Dynamin-2 | 23593 | | 1.37 |  |  |  |
| O35814 | Stress-induced-phosphoprotein 1 | 32579 | | 1.37 |  |  |  |
| Q08877 | Dynamin-3 | 21353 | | 1.36 |  |  |  |
| P02091 | Hemoglobin subunit beta-1 | 1999136 | | 1.36 |  |  |  |
| Q62745 | CD81 antigen | 40744 | | 1.34 |  |  |  |
| Q1WIM3 | Cell adhesion molecule 3 | 34953 | | 1.34 |  |  |  |
| Q66HA8 | Heat shock protein 105 kDa | 34031 | | 1.34 |  |  |  |
| O88778 | Protein bassoon | 17439 | | 1.34 |  |  |  |
| P97686 | Neuronal cell adhesion molecule | 24579 | | 1.33 |  |  |  |
| P05696 | Protein kinase C alpha type | 44086 | | 1.32 |  |  |  |
| P55068 | Brevican core protein | 31134 | | 1.30 |  |  |  |
| P16617 | Phosphoglycerate kinase 1 | 767554 | | 1.30 |  |  |  |
| Q68FY0 | Cytochrome b-c1 complex subunit 1, mitochondrial | 234561 | | 1.29 |  |  |  |
| P13264 | Glutaminase kidney isoform, mitochondrial | 43992 | | 1.29 |  |  |  |
| P32736 | Opioid-binding protein/cell adhesion molecule | 67662 | | 1.29 |  |  |  |
| P62963 | Profilin-1 | 218749 | | 1.29 |  |  |  |
| P51650 | Succinate-semialdehyde dehydrogenase, mitochondrial | 28507 | | 1.29 |  |  |  |
| P10860 | Glutamate dehydrogenase 1, mitochondrial | 180162 | | 1.28 |  |  |  |
| P85973 | Purine nucleoside phosphorylase | 74265 | | 1.28 |  |  |  |
| Q00981 | Ubiquitin carboxyl-terminal hydrolase isozyme L1 | 313039 | | 1.28 |  |  |  |
| P62815 | V-type proton ATPase subunit B, brain isoform | 209592 | | 1.28 |  |  |  |
| Q9WVC0 | Septin-7 | 395472 | | 1.27 |  |  |  |
| Q08163 | Adenylyl cyclase-associated protein 1 | 54071 | | 1.25 |  |  |  |
| P31596 | Excitatory amino acid transporter 2 | 629953 | | 1.25 |  |  |  |
| Q568Z9 | Phytanoyl-CoA hydroxylase-interacting protein | 50556 | | 1.25 |  |  |  |
| P19944 | 60S acidic ribosomal protein P1 | 104892 | | 1.24 |  |  |  |
| Q05140 | Clathrin coat assembly protein AP180 | 32000 | | 1.24 |  |  |  |
| P05065 | Fructose-bisphosphate aldolase A | 990686 | | 1.24 |  |  |  |
| Q9ESV6 | Glyceraldehyde-3-phosphate dehydrogenase, testis-specific | 44194 | | 1.24 |  |  |  |
| P84245 | Histone H3.3 | 127315 | | 1.24 |  |  |  |
| P68403 | Protein kinase C beta type | 53935 | | 1.24 |  |  |  |
| P48500 | Triosephosphate isomerase | 1300381 | | 1.24 |  |  |  |
| Q63028 | Alpha-adducin | 52970 | | 1.23 |  |  |  |
| P31399 | ATP synthase subunit d, mitochondrial | 302593 | | 1.23 |  |  |  |
| Q7M0E3 | Destrin | 85035 | | 1.23 |  |  |  |
| P63170 | Dynein light chain 1, cytoplasmic | 112119 | | 1.23 |  |  |  |
| P62804 | Histone H4 | 303780 | | 1.23 |  |  |  |
| P42123 | L-lactate dehydrogenase B chain | 329090 | | 1.23 |  |  |  |
| P81155 | Voltage-dependent anion-selective channel protein 2 | 271775 | | 1.23 |  |  |  |
| Q9QXQ0 | Alpha-actinin-4 | 55617 | | 1.22 |  |  |  |
| P15429 | Beta-enolase | 246104 | | 1.22 |  |  |  |
| P11442 | Clathrin heavy chain 1 | 267233 | | 1.22 |  |  |  |
| P07323 | Gamma-enolase | 1018573 | | 1.22 |  |  |  |
| P19234 | NADH dehydrogenase [ubiquinone] flavoprotein 2, mitochondrial | 65125 | | 1.22 |  |  |  |
| P48721 | Stress-70 protein, mitochondrial | 20669 | | 1.22 |  |  |  |
| P17764 | Acetyl-CoA acetyltransferase, mitochondrial | 168884 | | 1.20 |  |  |  |
| P04906 | Glutathione S-transferase P | 95313 | | 1.20 |  |  |  |
| P04631 | Protein S100-B | 259581 | | 1.20 |  |  |  |
| Q9Z2L0 | Voltage-dependent anion-selective channel protein 1 | 219826 | | 1.20 |  |  |  |
| Q8VHF5 | Citrate synthase, mitochondrial | 57729 | | 1.19 |  |  |  |
| P21575 | Dynamin-1 | 369766 | | 1.19 |  |  |  |
| P25286 | V-type proton ATPase 116 kDa subunit a1 | 73179 | | 1.19 |  |  |  |
| Q9WTT6 | Guanine deaminase | 49147 | | 1.18 |  |  |  |
| Q5XHZ0 | Heat shock protein 75 kDa, mitochondrial | 96537 | | 1.18 |  |  |  |
| Q6P7Q4 | Lactoylglutathione lyase | 190168 | | 1.18 |  |  |  |
| P15205 | Microtubule-associated protein 1B | 20798 | | 1.18 |  |  |  |
| Q5PQK1 | Septin-10 | 95480 | | 1.18 |  |  |  |
| P13086 | Succinate--CoA ligase [ADP/GDP-forming] subunit alpha, mitochondrial | 75671 | | 1.18 |  |  |  |
| P60881 | Synaptosomal-associated protein 25 | 480855 | | 1.18 |  |  |  |
| Q4V7C7 | Actin-related protein 3 | 115662 | | 1.17 |  |  |  |
| P54921 | Alpha-soluble NSF attachment protein | 66787 | | 1.17 |  |  |  |
| P04797 | Glyceraldehyde-3-phosphate dehydrogenase | 2486575 | | 1.17 |  |  |  |
| P52287 | Guanine nucleotide-binding protein G(I)/G(S)/G(T) subunit beta-3 | 86138 | | 1.17 |  |  |  |
| B3GNI6 | Septin-11 | 131405 | | 1.17 |  |  |  |
| P46462 | Transitional endoplasmic reticulum ATPase | 82988 | | 1.17 |  |  |  |
| Q5XI78 | 2-oxoglutarate dehydrogenase, mitochondrial | 36120 | | 1.16 |  |  |  |
| Q06647 | ATP synthase subunit O, mitochondrial | 378024 | | 1.16 |  |  |  |
| P12369 | cAMP-dependent protein kinase type II-beta regulatory subunit | 100767 | | 1.16 |  |  |  |
| P45592 | Cofilin-1 | 1483909 | | 1.16 |  |  |  |
| Q62950 | Dihydropyrimidinase-related protein 1 | 459470 | | 1.16 |  |  |  |
| P82995 | Heat shock protein HSP 90-alpha | 163807 | | 1.16 |  |  |  |
| Q00729 | Histone H2B type 1-A | 382720 | | 1.16 |  |  |  |
| Q99NA5 | Isocitrate dehydrogenase [NAD] subunit alpha, mitochondrial | 59238 | | 1.16 |  |  |  |
| Q68FX0 | Isocitrate dehydrogenase [NAD] subunit beta, mitochondrial | 56592 | | 1.16 |  |  |  |
| F1LRL9 | Microtubule-associated protein 1B | 20783 | | 1.16 |  |  |  |
| Q64548 | Reticulon-1 | 51572 | | 1.16 |  |  |  |
| P07632 | Superoxide dismutase [Cu-Zn] | 456499 | | 1.16 |  |  |  |
| P50554 | 4-aminobutyrate aminotransferase, mitochondrial | 83550 | | 1.15 |  |  |  |
| P85969 | Beta-soluble NSF attachment protein | 89797 | | 1.15 |  |  |  |
| Q6GMN2 | Brain-specific angiogenesis inhibitor 1-associated protein 2 | 25633 | | 1.15 |  |  |  |
| P12368 | cAMP-dependent protein kinase type II-alpha regulatory subunit | 44781 | | 1.15 |  |  |  |
| P07335 | Creatine kinase B-type | 1412008 | | 1.15 |  |  |  |
| P47819 | Glial fibrillary acidic protein | 364560 | | 1.15 |  |  |  |
| Q00715 | Histone H2B type 1 | 676576 | | 1.15 |  |  |  |
| Q9QYU4 | Ketimine reductase mu-crystallin | 57296 | | 1.15 |  |  |  |
| O88767 | Parkinson disease protein 7 homolog | 638775 | | 1.15 |  |  |  |
| P11598 | Protein disulfide-isomerase A3 | 34167 | | 1.15 |  |  |  |
| P26284 | Pyruvate dehydrogenase E1 component subunit alpha, somatic form, mitochondrial | 171944 | | 1.15 |  |  |  |
| Q6NYB7 | Ras-related protein Rab-1A | 184385 | | 1.15 |  |  |  |
| Q5BJU7 | Wiskott-Aldrich syndrome protein family member 1 | 57503 | | 1.15 |  |  |  |
| P04764 | Alpha-enolase | 651085 | | 1.13 |  |  |  |
| P10719 | ATP synthase subunit beta, mitochondrial | 1415358 | | 1.13 |  |  |  |
| P10818 | Cytochrome c oxidase subunit 6A1, mitochondrial | 77869 | | 1.13 |  |  |  |
| Q78P75 | Dynein light chain 2, cytoplasmic | 190743 | | 1.13 |  |  |  |
| B2RYW9 | Fumarylacetoacetate hydrolase domain-containing protein 2 | 35594 | | 1.13 |  |  |  |
| P63018 | Heat shock cognate 71 kDa protein | 788445 | | 1.13 |  |  |  |
| Q5RJQ4 | NAD-dependent protein deacetylase sirtuin-2 | 148305 | | 1.13 |  |  |  |
| Q561S0 | NADH dehydrogenase [ubiquinone] 1 alpha subcomplex subunit 10, mitochondrial | 177121 | | 1.13 |  |  |  |
| Q9Z0W5 | Protein kinase C and casein kinase substrate in neurons protein 1 | 74006 | | 1.13 |  |  |  |
| P11980 | Pyruvate kinase PKM | 904230 | | 1.13 |  |  |  |
| P63102 | 14-3-3 protein zeta/delta | 948023 | | 1.12 |  |  |  |
| P08081 | Clathrin light chain A | 44869 | | 1.12 |  |  |  |
| Q6P6R2 | Dihydrolipoyl dehydrogenase, mitochondrial | 109046 | | 1.12 |  |  |  |
| P08461 | Dihydrolipoyllysine-residue acetyltransferase component of pyruvate dehydrogenase complex, mitochondrial | 66979 | | 1.12 |  |  |  |
| Q62952 | Dihydropyrimidinase-related protein 3 | 117987 | | 1.12 |  |  |  |
| P14659 | Heat shock-related 70 kDa protein 2 | 291177 | | 1.12 |  |  |  |
| Q6AY09 | Heterogeneous nuclear ribonucleoprotein H2 | 86159 | | 1.12 |  |  |  |
| P41565 | Isocitrate dehydrogenase [NAD] subunit gamma 1, mitochondrial | 80897 | | 1.12 |  |  |  |
| P04636 | Malate dehydrogenase, mitochondrial | 1998639 | | 1.12 |  |  |  |
| O08839 | Myc box-dependent-interacting protein 1 | 145776 | | 1.12 |  |  |  |
| Q66HF1 | NADH-ubiquinone oxidoreductase 75 kDa subunit, mitochondrial | 83136 | | 1.12 |  |  |  |
| P10111 | Peptidyl-prolyl cis-trans isomerase A | 2234961 | | 1.12 |  |  |  |
| P31044 | Phosphatidylethanolamine-binding protein 1 | 1311468 | | 1.12 |  |  |  |
| P49432 | Pyruvate dehydrogenase E1 component subunit beta, mitochondrial | 254130 | | 1.12 |  |  |  |
| P62944 | AP-2 complex subunit beta | 22433 | | 1.11 |  |  |  |
| P25809 | Creatine kinase U-type, mitochondrial | 314535 | | 1.11 |  |  |  |
| Q5RKI1 | Eukaryotic initiation factor 4A-II | 70351 | | 1.11 |  |  |  |
| Q8VHV7 | Heterogeneous nuclear ribonucleoprotein H | 82884 | | 1.11 |  |  |  |
| P15146 | Microtubule-associated protein 2 | 125203 | | 1.11 |  |  |  |
| P16884 | Neurofilament heavy polypeptide | 76736 | | 1.11 |  |  |  |
| P21707 | Synaptotagmin-1 | 211339 | | 1.11 |  |  |  |
| P61265 | Syntaxin-1B | 107163 | | 1.11 |  |  |  |
| P37805 | Transgelin-3 | 126322 | | 1.11 |  |  |  |
| D3ZQL7 | Tubulin polymerization-promoting protein | 112468 | | 1.11 |  |  |  |
| P68255 | 14-3-3 protein theta | 413644 | | 1.10 |  |  |  |
| P13221 | Aspartate aminotransferase, cytoplasmic | 629234 | | 1.10 |  |  |  |
| P21571 | ATP synthase-coupling factor 6, mitochondrial | 134484 | | 1.10 |  |  |  |
| P20788 | Cytochrome b-c1 complex subunit Rieske, mitochondrial | 169217 | | 1.10 |  |  |  |
| P85834 | Elongation factor Tu, mitochondrial | 72074 | | 1.10 |  |  |  |
| P54313 | Guanine nucleotide-binding protein G(I)/G(S)/G(T) subunit beta-2 | 367556 | | 1.10 |  |  |  |
| O35353 | Guanine nucleotide-binding protein subunit beta-4 | 470315 | | 1.10 |  |  |  |
| P67779 | Prohibitin | 71500 | | 1.10 |  |  |  |
| P13668 | Stathmin | 233059 | | 1.10 |  |  |  |
| O08838 | Amphiphysin | 23595 | | 1.09 |  |  |  |
| Q03344 | ATPase inhibitor, mitochondrial | 149750 | | 1.09 |  |  |  |
| P62959 | Histidine triad nucleotide-binding protein 1 | 188319 | | 1.09 |  |  |  |
| P07936 | Neuromodulin | 262389 | | 1.09 |  |  |  |
| Q05982 | Nucleoside diphosphate kinase A | 552315 | | 1.09 |  |  |  |
| Q5XIH7 | Prohibitin-2 | 56926 | | 1.09 |  |  |  |
| Q05962 | ADP/ATP translocase 1 | 221266 | | 1.08 |  |  |  |
| P18418 | Calreticulin | 45318 | | 1.08 |  |  |  |
| Q5M7A7 | CB1 cannabinoid receptor-interacting protein 1 | 292215 | | 1.08 |  |  |  |
| P35704 | Peroxiredoxin-2 | 292191 | | 1.08 |  |  |  |
| Q9R063 | Peroxiredoxin-5, mitochondrial | 334343 | | 1.08 |  |  |  |
| P22062 | Protein-L-isoaspartate(D-aspartate) O-methyltransferase | 81446 | | 1.08 |  |  |  |
| P50137 | Transketolase | 151414 | | 1.08 |  |  |  |
| P68511 | 14-3-3 protein eta | 424224 | | 1.07 |  |  |  |
| Q5M9I5 | Cytochrome b-c1 complex subunit 6, mitochondrial | 152022 | | 1.07 |  |  |  |
| P11240 | Cytochrome c oxidase subunit 5A, mitochondrial | 676491 | | 1.07 |  |  |  |
| P62632 | Elongation factor 1-alpha 2 | 251587 | | 1.07 |  |  |  |
| O35179 | Endophilin-A1 | 289316 | | 1.07 |  |  |  |
| P06761 | Endoplasmic reticulum chaperone BiP | 90695 | | 1.07 |  |  |  |
| P50399 | Rab GDP dissociation inhibitor beta | 506040 | | 1.07 |  |  |  |
| Q63537 | Synapsin-2 | 437020 | | 1.07 |  |  |  |
| Q05546 | Tenascin-R | 34169 | | 1.07 |  |  |  |
| Q5XIF6 | Tubulin alpha-4A chain | 2517133 | | 1.07 |  |  |  |
| P26772 | 10 kDa heat shock protein, mitochondrial | 175860 | | 1.06 |  |  |  |
| P35213 | 14-3-3 protein beta/alpha | 463950 | | 1.06 |  |  |  |
| P62630 | Elongation factor 1-alpha 1 | 267173 | | 1.06 |  |  |  |
| P09117 | Fructose-bisphosphate aldolase C | 638096 | | 1.06 |  |  |  |
| P54311 | Guanine nucleotide-binding protein G(I)/G(S)/G(T) subunit beta-1 | 824798 | | 1.06 |  |  |  |
| P06685 | Sodium/potassium-transporting ATPase subunit alpha-1 | 432628 | | 1.06 |  |  |  |
| P09951 | Synapsin-1 | 678733 | | 1.06 |  |  |  |
| Q68FR8 | Tubulin alpha-3 chain | 2258392 | | 1.06 |  |  |  |
| P61983 | 14-3-3 protein gamma | 659437 | | 1.05 |  |  |  |
| P23565 | Alpha-internexin | 572892 | | 1.05 |  |  |  |
| P37377 | Alpha-synuclein | 728669 | | 1.05 |  |  |  |
| P15999 | ATP synthase subunit alpha, mitochondrial | 725191 | | 1.05 |  |  |  |
| P34058 | Heat shock protein HSP 90-beta | 172695 | | 1.05 |  |  |  |
| P34926 | Microtubule-associated protein 1A | 45150 | | 1.05 |  |  |  |
| P06687 | Sodium/potassium-transporting ATPase subunit alpha-3 | 605244 | | 1.05 |  |  |  |
| Q4QRB4 | Tubulin beta-3 chain | 3090929 | | 1.05 |  |  |  |
| P12839 | Neurofilament medium polypeptide | 250801 | | 1.04 |  |  |  |
| P62260 | 14-3-3 protein epsilon | 462696 | | 1.03 |  |  |  |
| P68136 | Actin, alpha skeletal muscle | 1361052 | | 1.03 |  |  |  |
| P62738 | Actin, aortic smooth muscle | 1365534 | | 1.03 |  |  |  |
| P63269 | Actin, gamma-enteric smooth muscle | 1365534 | | 1.03 |  |  |  |
| Q6AY56 | Tubulin alpha-8 chain | 1250953 | | 1.03 |  |  |  |
| P29476 | Nitric oxide synthase, brain | 22711 | | -0.16 |  |  |  |
| Q769K2 | N-acyl-phosphatidylethanolamine-hydrolyzing phospholipase D | 24670 | | -0.53 |  |  |  |
| P04182 | Ornithine aminotransferase, mitochondrial | 31765 | | -0.59 |  |  |  |
| P00564 | Creatine kinase M-type | 21709 | | -0.67 |  |  |  |
| P12346 | Serotransferrin | 21449 | | -0.69 |  |  |  |
| P03994 | Hyaluronan and proteoglycan link protein 1 | 35500 | | -0.71 |  |  |  |
| Q6P502 | T-complex protein 1 subunit gamma | 79099 | | -0.71 |  |  |  |
| Q9WVB1 | Ras-related protein Rab-6A | 66640 | | -0.76 |  |  |  |
| P11951 | Cytochrome c oxidase subunit 6C-2 | 300282 | | -0.79 |  |  |  |
| P51156 | Ras-related protein Rab-26 | 111428 | | -0.80 |  |  |  |
| P51146 | Ras-related protein Rab-4B | 113819 | | -0.80 |  |  |  |
| Q53B90 | Ras-related protein Rab-43 | 126786 | | -0.81 |  |  |  |
| P61589 | Transforming protein RhoA | 147558 | | -0.82 |  |  |  |
| P02770 | Albumin | 188167 | | -0.83 |  |  |  |
| Q8VBU2 | Protein NDRG2 | 53686 | | -0.84 |  |  |  |
| Q5RKI0 | WD repeat-containing protein 1 | 38302 | | -0.85 |  |  |  |
| P08413 | Calcium/calmodulin-dependent protein kinase type II subunit beta | 599535 | | -0.86 |  |  |  |
| P0DP29 | Calmodulin-1 | 616733 | | -0.86 |  |  |  |
| P0DP30 | Calmodulin-2 | 616733 | | -0.86 |  |  |  |
| P0DP31 | Calmodulin-3 | 616733 | | -0.86 |  |  |  |
| Q62658 | Peptidyl-prolyl cis-trans isomerase FKBP1A | 315975 | | -0.88 |  |  |  |
| P61107 | Ras-related protein Rab-14 | 198451 | | -0.88 |  |  |  |
| Q62910 | Synaptojanin-1 | 15267 | | -0.88 |  |  |  |
| Q63942 | GTP-binding protein Rab-3D | 184649 | | -0.91 |  |  |  |
| P59215 | Guanine nucleotide-binding protein G(o) subunit alpha | 874241 | | -0.92 |  |  |  |
| P13233 | 2',3'-cyclic-nucleotide 3'-phosphodiesterase | 1135655 | | -0.93 |  |  |  |
| P11275 | Calcium/calmodulin-dependent protein kinase type II subunit alpha | 1310207 | | -0.93 |  |  |  |
| P09606 | Glutamine synthetase | 520869 | | -0.93 |  |  |  |
| P11730 | Calcium/calmodulin-dependent protein kinase type II subunit gamma | 391094 | | -0.94 |  |  |  |
| P60711 | Actin, cytoplasmic 1 | 3183498 | | -0.95 |  |  |  |
| Q63754 | Beta-synuclein | 1326910 | | -0.96 |  |  |  |
| P60203 | Myelin proteolipid protein | 1254990 | | -0.97 |  |  |  |
| P68370 | Tubulin alpha-1A chain | 2801302 | | -0.98 |  |  |  |
| Q6P9T8 | Tubulin beta-4B chain | 4112240 | | -0.99 |  |  |  |
| ^a^Accesion ID from uniprot.org database; Negative and positive values of fold change represent down-regulation and up-regulation, respectively, in Pb group when compared to control group. | | | | | | |  |
|  |  |  |  |  |  |  |  |

| Table S3. Global proteomic profile of rats motor cortex exposed to lead acetate in comparison to control group. List of proteins with exclusive expression in one of the groups. | | | |  |
| --- | --- | --- | --- | --- |
|  |  |  |  |  |
| Accession Idª | **Protein Description** | **Score** | **Unique** |  |
| P10687 | 1-phosphatidylinositol 4,5-bisphosphate phosphodiesterase beta-1 | 16481 | Ctl |  |
| O88761 | 26S proteasome non-ATPase regulatory subunit 1 | 18942 | Pb |  |
| Q63569 | 26S proteasome regulatory subunit 6A | 26545 | Ctl |  |
| P22071 | 3 beta-hydroxysteroid dehydrogenase/Delta 5-->4-isomerase type 1 | 49662 | Ctl |  |
| Q62878 | 3 beta-hydroxysteroid dehydrogenase/Delta 5-->4-isomerase type 4 | 27809 | Ctl |  |
| P21775 | 3-ketoacyl-CoA thiolase A, peroxisomal | 24459 | Pb |  |
| P38983 | 40S ribosomal protein SA | 18100 | Pb |  |
| Q91ZS3 | 45 kDa calcium-binding protein | 40241 | Pb |  |
| P19945 | 60S acidic ribosomal protein P0 | 28245 | Ctl |  |
| P27213 | 6-pyruvoyl tetrahydrobiopterin synthase | 112703 | Ctl |  |
| P33436 | 72 kDa type IV collagenase | 18911 | Ctl |  |
| P37136 | Acetylcholinesterase | 28470 | Pb |  |
| Q5XI22 | Acetyl-CoA acetyltransferase, cytosolic | 22393 | Ctl |  |
| Q6SKG1 | Acyl-coenzyme A synthetase ACSM3, mitochondrial | 16807 | Pb |  |
| Q63768 | Adapter molecule crk | 29823 | Ctl |  |
| Q7TP48 | Adipocyte plasma membrane-associated protein | 35218 | Pb |  |
| P0C6C0 | A-kinase anchor protein SPHKAP | 21504 | Ctl |  |
| P50475 | Alanine--tRNA ligase, cytoplasmic | 21792 | Ctl |  |
| P00330 | Alcohol dehydrogenase 1 | 243343 | Ctl |  |
| Q62994 | Alpha-(1,3)-fucosyltransferase 4 | 22573 | Ctl |  |
| P52303 | AP-1 complex subunit beta-1 | 20441 | Pb |  |
| P0C1X8 | AP2-associated protein kinase 1 | 39344 | Ctl |  |
| P02650 | Apolipoprotein E | 28351 | Ctl |  |
| P29419 | ATP synthase subunit e, mitochondrial | 96917 | Ctl |  |
| Q9WTP0 | Band 4.1-like protein 1 | 19600 | Ctl |  |
| P26453 | Basigin | 28981 | Ctl |  |
| P53563 | Bcl-2-like protein 1 | 33980 | Pb |  |
| Q02527 | Beta-1,4-mannosyl-glycoprotein 4-beta-N-acetylglucosaminyltransferase | 47812 | Pb |  |
| Q63276 | Bile acid-CoA:amino acid N-acyltransferase | 40352 | Pb |  |
| Q68FX6 | Calcium-binding and spermatid-specific protein 1 | 26374 | Ctl |  |
| O88751 | Calcium-binding protein 1 | 38934 | Ctl |  |
| Q62717 | Calcium-dependent secretion activator 1 | 21859 | Pb |  |
| P63055 | Calmodulin regulator protein PCP4 | 297094 | Ctl |  |
| Q78EJ9 | Calpain-8 | 37563 | Ctl |  |
| P27791 | cAMP-dependent protein kinase catalytic subunit alpha | 15514 | Ctl |  |
| P68182 | cAMP-dependent protein kinase catalytic subunit beta | 31057 | Ctl |  |
| Q8CHI9 | Carbohydrate sulfotransferase 15 | 57281 | Ctl |  |
| P67874 | Casein kinase II subunit beta | 46801 | Ctl |  |
| P04762 | Catalase | 21520 | Ctl |  |
| Q9WU82 | Catenin beta-1 | 34651 | Ctl |  |
| Q9R1T3 | Cathepsin Z | 35300 | Ctl |  |
| P97586 | Cell growth regulator with EF hand domain protein 1 | 36459 | Pb |  |
| Q62865 | cGMP-inhibited 3',5'-cyclic phosphodiesterase A | 16382 | Ctl |  |
| B1WC61 | Complex I assembly factor ACAD9, mitochondrial | 25508 | Ctl |  |
| O09018 | COUP transcription factor 2 | 33525 | Pb |  |
| P09605 | Creatine kinase S-type, mitochondrial | 16680 | Ctl |  |
| Q9Z2F5 | C-terminal-binding protein 1 | 26392 | Ctl |  |
| Q5FVM5 | Cyclic AMP-responsive element-binding protein 3-like protein 3 | 34941 | Pb |  |
| P14841 | Cystatin-C | 103532 | Pb |  |
| Q5PQS5 | Cysteine and tyrosine-rich protein 1 | 36920 | Pb |  |
| Q9QXU8 | Cytoplasmic dynein 1 light intermediate chain 1 | 24067 | Ctl |  |
| Q6Q0N1 | Cytosolic non-specific dipeptidase | 26490 | Ctl |  |
| Q63342 | Dimethylglycine dehydrogenase, mitochondrial | 20613 | Pb |  |
| Q566C7 | Diphosphoinositol polyphosphate phosphohydrolase 1 | 32803 | Ctl |  |
| O88797 | Disabled homolog 2 | 22669 | Ctl |  |
| Q9ESW0 | DNA damage-binding protein 1 | 17093 | Pb |  |
| P07153 | Dolichyl-diphosphooligosaccharide--protein glycosyltransferase subunit 1 | 16756 | Ctl |  |
| P36506 | Dual specificity mitogen-activated protein kinase kinase 2 | 27068 | Ctl |  |
| B4F766 | Dymeclin | 24203 | Pb |  |
| Q6P6T4 | Echinoderm microtubule-associated protein-like 2 | 20734 | Pb |  |
| Q4FZY0 | EF-hand domain-containing protein D2 | 51103 | Ctl |  |
| Q641Z6 | EH domain-containing protein 1 | 24025 | Pb |  |
| Q5XIQ2 | ELMO domain-containing protein 3 | 36566 | Ctl |  |
| Q9QYU2 | Elongation factor Ts, mitochondrial | 17315 | Ctl |  |
| P14604 | Enoyl-CoA hydratase, mitochondrial | 53210 | Pb |  |
| P56571 | ES1 protein homolog, mitochondrial | 40189 | Pb |  |
| Q6P9U8 | Eukaryotic translation initiation factor 3 subunit H | 32978 | Ctl |  |
| Q3T1J1 | Eukaryotic translation initiation factor 5A-1 | 26920 | Pb |  |
| P11762 | Galectin-1 | 40259 | Pb |  |
| P36375 | Glandular kallikrein-10 | 35068 | Pb |  |
| Q05683 | Glutamate decarboxylase 2 | 22914 | Ctl |  |
| Q9QWE9 | Glutathione hydrolase 5 proenzyme | 104059 | Ctl |  |
| P08010 | Glutathione S-transferase Mu 2 | 19867 | Ctl |  |
| Q5I0P2 | Glycine cleavage system H protein, mitochondrial | 74188 | Pb |  |
| Q6AYF6 | Glycosyltransferase 8 domain-containing protein 1 | 34543 | Pb |  |
| Q5I0D1 | Glyoxalase domain-containing protein 4 | 22447 | Pb |  |
| Q5FVG8 | GRAM domain-containing protein 2B | 24940 | Ctl |  |
| Q9JHZ4 | GRIP1-associated protein 1 | 30243 | Pb |  |
| P62994 | Growth factor receptor-bound protein 2 | 22511 | Ctl |  |
| P20171 | GTPase HRas | 35763 | Pb |  |
| P82471 | Guanine nucleotide-binding protein G(q) subunit alpha | 26064 | Ctl |  |
| Q811S9 | Guanine nucleotide-binding protein-like 3 | 30233 | Ctl |  |
| O35162 | Heat shock 70 kDa protein 13 | 27554 | Pb |  |
| O35543 | Hematopoietic prostaglandin D synthase | 30204 | Ctl |  |
| F1LQ48 | Heterogeneous nuclear ribonucleoprotein L | 61930 | Pb |  |
| Q6IMY8 | Heterogeneous nuclear ribonucleoprotein U | 19283 | Ctl |  |
| A6YP92 | Homeobox protein ARX | 16553 | Pb |  |
| Q63692 | Hsp90 co-chaperone Cdc37 | 20294 | Ctl |  |
| P17425 | Hydroxymethylglutaryl-CoA synthase, cytoplasmic | 28932 | Ctl |  |
| Q6IE24 | Inactive ubiquitin carboxyl-terminal hydrolase 54 | 13309 | Pb |  |
| Q8CGX0 | Insulin-like growth factor 2 mRNA-binding protein 1 | 20045 | Ctl |  |
| Q99MF5 | Interleukin-11 | 28873 | Pb |  |
| Q8VI04 | Isoaspartyl peptidase/L-asparaginase | 24720 | Ctl |  |
| P00758 | Kallikrein-1 | 23018 | Pb |  |
| F1LZF0 | Kelch-like protein 2 | 21305 | Pb |  |
| Q6IFU7 | Keratin, type I cytoskeletal 42 | 16590 | Pb |  |
| Q6IG02 | Keratin, type II cytoskeletal 2 epidermal | 20349 | Ctl |  |
| Q6IG00 | Keratin, type II cytoskeletal 4 | 23796 | Ctl |  |
| Q6IG05 | Keratin, type II cytoskeletal 75 | 23796 | Ctl |  |
| Q9EQG6 | Kinase D-interacting substrate of 220 kDa | 17935 | Ctl |  |
| Q811X6 | Lambda-crystallin homolog | 28939 | Pb |  |
| B1H234 | Leucine-rich repeat transmembrane protein FLRT3 | 22658 | Pb |  |
| Q62813 | Limbic system-associated membrane protein | 23305 | Ctl |  |
| P18163 | Long-chain-fatty-acid--CoA ligase 1 | 19887 | Ctl |  |
| Q00495 | Macrophage colony-stimulating factor 1 receptor | 14019 | Pb |  |
| Q5XII0 | Mammalian ependymin-related protein 1 | 26683 | Pb |  |
| P43244 | Matrin-3 | 25272 | Pb |  |
| O88382 | Membrane-associated guanylate kinase, WW and PDZ domain-containing protein 2 | 56686 | Pb |  |
| Q5U2R0 | Methionine adenosyltransferase 2 subunit beta | 39814 | Pb |  |
| Q66HR2 | Microtubule-associated protein RP/EB family member 1 | 72837 | Pb |  |
| Q6XVN8 | Microtubule-associated proteins 1A/1B light chain 3A | 18456 | Pb |  |
| P21708 | Mitogen-activated protein kinase 3 | 21334 | Pb |  |
| P27704 | Mitogen-activated protein kinase 6 | 13123 | Pb |  |
| Q5M820 | MORF4 family-associated protein 1 | 207368 | Pb |  |
| Q9R1J4 | Myocilin | 34984 | Pb |  |
| P18666 | Myosin regulatory light chain 12B | 63641 | Ctl |  |
| P13832 | Myosin regulatory light chain RLC-A | 63641 | Ctl |  |
| P62775 | Myotrophin | 81977 | Pb |  |
| Q05695 | Neural cell adhesion molecule L1 | 17999 | Ctl |  |
| Q812E9 | Neuronal membrane glycoprotein M6-a | 85809 | Ctl |  |
| Q9JJK1 | Neuronal membrane glycoprotein M6-b | 36981 | Ctl |  |
| Q5XIA1 | Nicalin | 17167 | Ctl |  |
| D3ZSK5 | N-lysine methyltransferase SETD6 | 23339 | Ctl |  |
| O09017 | Nuclear receptor subfamily 2 group F member 6 | 28713 | Pb |  |
| Q9QXL7 | Nucleoside diphosphate kinase 7 | 19932 | Ctl |  |
| Q4R180 | Origin recognition complex subunit 3 | 18674 | Ctl |  |
| Q63716 | Peroxiredoxin-1 | 43811 | Ctl |  |
| Q9Z0V5 | Peroxiredoxin-4 | 30384 | Ctl |  |
| P04176 | Phenylalanine-4-hydroxylase | 22430 | Ctl |  |
| Q9R0I8 | Phosphatidylinositol 5-phosphate 4-kinase type-2 alpha | 37851 | Pb |  |
| Q64542 | Plasma membrane calcium-transporting ATPase 4 | 21600 | Ctl |  |
| Q9WV25 | Poly(U)-binding-splicing factor PUF60 | 23598 | Ctl |  |
| Q6Q1P3 | Potassium channel subfamily K member 18 | 36796 | Pb |  |
| P04094 | Proenkephalin-A | 21909 | Ctl |  |
| P05710 | Prolactin receptor | 27499 | Pb |  |
| Q56B11 | Proline-, glutamic acid- and leucine-rich protein 1 | 15308 | Pb |  |
| P10960 | Prosaposin | 37489 | Ctl |  |
| Q5XIU5 | Proteasome inhibitor PI31 subunit | 41737 | Pb |  |
| P18421 | Proteasome subunit beta type-1 | 44465 | Ctl |  |
| Q9Z250 | Protein lin-7 homolog A | 62130 | Pb |  |
| Q792I0 | Protein lin-7 homolog C | 62130 | Pb |  |
| F1LNI5 | Protein phosphatase 1G | 31869 | Ctl |  |
| Q5XFW8 | Protein SEC13 homolog | 32074 | Pb |  |
| P23999 | Protein S-Myc | 31016 | Ctl |  |
| P0C5X8 | Protein tweety homolog 1 | 36496 | Pb |  |
| Q63132 | Proto-oncogene tyrosine-protein kinase ROS | 13123 | Pb |  |
| O54880 | Rab effector Noc2 | 26129 | Pb |  |
| O55007 | Ral GTPase-activating protein subunit alpha-1 | 32502 | Pb |  |
| Q9JK11 | Reticulon-4 | 42583 | Pb |  |
| Q920A6 | Retinoid-inducible serine carboxypeptidase | 16171 | Ctl |  |
| P62747 | Rho-related GTP-binding protein RhoB | 58818 | Ctl |  |
| Q3B7K9 | RUN domain-containing protein 3B | 26530 | Ctl |  |
| P11507 | Sarcoplasmic/endoplasmic reticulum calcium ATPase 2 | 22909 | Ctl |  |
| P56603 | Secretory carrier-associated membrane protein 1 | 31400 | Ctl |  |
| P14056 | Serine/threonine-protein kinase A-Raf | 19986 | Ctl |  |
| D3ZSZ3 | Serine/threonine-protein kinase NLK | 33205 | Ctl |  |
| Q9R011 | Serine/threonine-protein kinase PLK3 | 21304 | Ctl |  |
| Q4QQT4 | Serine/threonine-protein phosphatase 2A 65 kDa regulatory subunit A beta isoform | 19664 | Ctl |  |
| P63331 | Serine/threonine-protein phosphatase 2A catalytic subunit alpha isoform | 37546 | Pb |  |
| P62716 | Serine/threonine-protein phosphatase 2A catalytic subunit beta isoform | 37546 | Pb |  |
| P62138 | Serine/threonine-protein phosphatase PP1-alpha catalytic subunit | 28843 | Ctl |  |
| P62142 | Serine/threonine-protein phosphatase PP1-beta catalytic subunit | 24015 | Ctl |  |
| P63088 | Serine/threonine-protein phosphatase PP1-gamma catalytic subunit | 16524 | Ctl |  |
| P09139 | Serine--pyruvate aminotransferase, mitochondrial | 32981 | Ctl |  |
| P70564 | Serpin B5 | 48837 | Ctl |  |
| Q63965 | Sideroflexin-1 | 38041 | Pb |  |
| Q9JHY2 | Sideroflexin-3 | 25636 | Pb |  |
| Q4FZX7 | Signal recognition particle receptor subunit beta | 22388 | Pb |  |
| Q07490 | Signal transducer CD24 | 115679 | Ctl |  |
| P61959 | Small ubiquitin-related modifier 2 | 34688 | Ctl |  |
| Q5XIF4 | Small ubiquitin-related modifier 3 | 34688 | Ctl |  |
| P55018 | Solute carrier family 12 member 3 | 26330 | Pb |  |
| Q07647 | Solute carrier family 2, facilitated glucose transporter member 3 | 23104 | Pb |  |
| Q9WTW8 | Solute carrier family 23 member 2 | 16098 | Pb |  |
| Q6AXV4 | Sorting and assembly machinery component 50 homolog | 14225 | Pb |  |
| Q6PEC4 | S-phase kinase-associated protein 1 | 110809 | Ctl |  |
| Q66HL2 | Src substrate cortactin | 18613 | Ctl |  |
| P07152 | Stromelysin-2 | 32824 | Pb |  |
| G3V9R3 | Sulfotransferase 1 family member D1 | 28831 | Pb |  |
| Q62876 | Synaptogyrin-1 | 49012 | Ctl |  |
| Q4KLN0 | Syntenin-2 | 23869 | Pb |  |
| Q5CD77 | TBC1 domain family member 14 | 39207 | Pb |  |
| Q5BK49 | T-cell surface protein tactile | 25570 | Pb |  |
| P28480 | T-complex protein 1 subunit alpha | 25326 | Ctl |  |
| Q5XIM9 | T-complex protein 1 subunit beta | 29563 | Pb |  |
| Q5XHX9 | Tesmin | 26662 | Ctl |  |
| Q4QQS2 | Tetratricopeptide repeat protein 30A2 | 27360 | Ctl |  |
| Q6P3V7 | Tetratricopeptide repeat protein 41 | 22122 | Pb |  |
| A2RUW1 | Toll-interacting protein | 44877 | Ctl |  |
| P63029 | Translationally-controlled tumor protein | 28388 | Ctl |  |
| P62603 | Tripartite motif-containing protein 26 | 39648 | Ctl |  |
| A8WCF8 | Tumor protein p63-regulated gene 1-like protein | 50119 | Pb |  |
| P04177 | Tyrosine 3-monooxygenase | 20904 | Ctl |  |
| Q64725 | Tyrosine-protein kinase SYK | 30453 | Ctl |  |
| Q9EQX9 | Ubiquitin-conjugating enzyme E2 N | 40237 | Ctl |  |
| Q7M767 | Ubiquitin-conjugating enzyme E2 variant 2 | 83742 | Pb |  |
| Q6AY39 | UDP-GalNAc:beta-1,3-N-acetylgalactosaminyltransferase 1 | 61747 | Ctl |  |
| Q9Z270 | Vesicle-associated membrane protein-associated protein A | 34231 | Pb |  |
| Q5GH56 | XK-related protein 7 | 25214 | Ctl |  |
| Q6QIX3 | Zinc transporter 3 | 25918 | Ctl |  |
| ^a^Accesion ID from uniprot.org database; Proteins with unique expression in control group (Ctl) and in lead acetate-exposed group (Pb). | | | |  |
|  |  |  |  |  |
